# Connecting the dots: Secondary science teacher candidates’ uptake of the core practice of facilitating sensemaking discussions from teacher education experiences

**DOI:** 10.1016/j.tate.2019.01.006

**Published:** 2019-04

**Authors:** Matthew Kloser, Matthew Wilsey, Tia C. Madkins, Mark Windschitl

**Affiliations:** a University of Notre Dame, Institute of Educational Initiatives, 107 Carole Sandner Hall, Notre Dame, IN, 46556, USA; b University of Washington, College of Education, 115B Miller Hall, Seattle, WA, 98195, USA

## Abstract

• Rehearsals of teaching practice function as a bridge from methods to the classroom.
• Organizational tools help increase the visual representations during discussions.
• Elements of framing and closing discussions are taken up less to the classroom.
• Talk moves are taken up extensively in rehearsals and the classroom.
• Explicit representations of practice are most often taken up by novice teachers.

Preparing pre-service teacher candidates to begin developing expertise in complex instructional practices has been addressed by an increasing number of teacher educators through practice-based teacher education. At its best, practice-based teacher education provides teacher candidates with learning opportunities that reflect the authentic work of teaching, including its complexities, and draws upon the professional knowledge and judgment of the teacher needed to address students' needs (Grossman, Kavanagh, & Dean, 2018; Zeichner, 2012). However, critiques of practice-based teacher education and its focus on core practices caution against the possible regression of instruction away from a responsive and flexible act toward overly prescribed, decontextualized performance (Zeichner, 2012). Such regression could result in two problems for classrooms: a) the tendency for novice teachers to enact scripted, robotic-like instruction that is divorced from professional judgment (Grossman et al., 2018); and b) the potential for novice teachers to enact practices that do not attend to the diverse cultural, social, and intellectual needs of students which would, in turn, exacerbate, rather than reduce, inequities among students’ learning opportunities in the classroom (Zeichner, 2012).

The preparation of novice science teachers to facilitate sensemaking discussions presents an interesting case to explore aspects of practice-based teacher education for several key reasons. First, discussion is central to science learning. Nearly thirty years ago, Lemke (1990) argued that science is a social process that is both conducted and understood through talk. For young people, making sense of the natural world requires understanding the major accounts of science by doing science, and doing science requires that students engage in the practice of communicating and negotiating ideas. More recent national standards (NGSS Lead States, 2013) reinforce the importance of student talk—that it is necessary for the construction of and deliberations about models, arguments, and explanations.

In addition, the focus on this practice presents opportunities to think about novice teacher learning in light of the two potential problems of a practice-based approach – the potential for reducing the practice to a merely technical act and the potential for exacerbating inequities within the classroom. First, we conceptualize the facilitation of science sensemaking discussions as a complex practice that stands in contrast to potentially isolated skills like “asking questions”. By our definition, the former responds to students’ existing and emerging ideas that can be used to construct collective understanding and, therefore, cannot be reduced to a pre-defined sequence of technical steps. This is not to say that novice teachers will not struggle engaging in this instructional practice. Indeed, facilitating such meaningful conversations is not innate for novice teachers, nor will they simply “grow into” these skills without guidance and coaching (Ball & Forzani, 2009). Still, the practice of facilitating sensemaking discussions contrasts discrete instructional skills, like class questioning, that can follow a rote pattern like triadic dialogue (Resnick, Asterhan, & Clarke, 2015) or exist as a parade of low-level, teacher-focused questioning that is more mechanical in nature (Banilower, Smith, Weiss, & Pasley, 2006; Weiss, Pasley, Smith, Banilower, & Heck, 2003).

Second, as is discussed more in-depth below, the conceptualization of facilitating sensemaking discussions observed in this study explicitly is seen as a means for drawing on students’ funds of knowledge (González, Moll, & Amanti, 2005), using their ways of knowing to create collective meaning, and making explicit the ways in which scientists engage in discussion, debate, and justification (Windschitl, Thompson, & Braaten, 2018). Thus, investigating the practice of facilitating sensemaking discussions in science classrooms provides a window into how complex practice is decomposed and taken up among novice teachers and how the framework for equity that conceptually undergirds the practice does or does not get taken up by teacher candidates when working with students.

Specifically, this study uses two case studies from university-based teacher education to better understand what elements of the practice of facilitating sensemaking discussions are taken up by science teacher candidates from their practice-based science methods courses and enacted in their own instruction. The goal is to first identify what constitutive elements of the core practice are negotiated – both implicitly and explicitly – in the methods courses. Mapping out these elements and dispositions will illuminate how teacher educators organize the complexities of the practice that enable its flexible use to meet the needs of all learners. We then ‘connect the dots’ between what occurs in a science methods course and what teacher candidates enact both proximally in rehearsals – an approximation of practice in which the teacher candidate acts as the teacher among peers and a teacher educator (Davis et al., 2017) – and more distally in classrooms with students. Our research questions explore:

1. How are frameworks for the core practice of facilitating sensemaking discussions in science classrooms similar and different across two university-based teacher education programs?
2. Within and across each program, what elements of the practice do teacher candidates take up when:
  a. Facilitating a discussion with peers during a rehearsal, and
  b. Facilitating a discussion with students in an authentic classroom environment?
3. How do teacher candidates' uptake of the practice in rehearsals and classroom discussions vary based on whether elements of the practice are presented implicitly or explicitly in the methods course?

To address these questions, we discuss the role of practice-based teacher education and outline our conception of sensemaking discussion in science classrooms that promote ‘ambitious’ (Windschitl, Thompson, Braaten, & Stroupe, 2012) learning environments centered on student thinking. We then describe our method for developing frameworks for the teaching of this core practice in two university settings that serve as the analytical structure for ‘connecting the dots’ between what was experienced in the science methods course and what was reflected in both the rehearsals and the classroom discussions from eight teacher candidates. Finally, we discuss the implications of the results for whether, when, and how teacher candidates take up the practice of facilitating sensemaking discussions.

## 1 Background literature and conceptual framing

### 1.1 Core practices as a response to persistent challenges in teacher education

This study focuses on two features of teacher preparation – a disciplinary methods course focused on core practices, and the use of rehearsal as a teacher education pedagogy. Practices are recurring professional work that includes aspects of planning, enactment, or reflection. They are comprised of strategies, moves, and tools used by teachers to achieve particular learning goals (Ball, Sleep, Boerst, & Bass, 2009). Core practices refer to “specific, routine aspects of teaching that demand the exercise of professional judgment and the creation of meaningful intellectual and social community for teachers, teacher educators, and students” (McDonald, Kazemi, & Kavanagh, 2013, p. 378). These are considered ‘core’ in the sense that, given limited time and resources, particular practices are more central to achieving disciplinary learning goals through student-centered instruction. It is important to note that practices are *not* scripts, competencies, or behaviors, rather, they are thought of more expansively:

• They follow prototypical (but adaptable) sequences of activity in which teachers interact with learners.
• They adhere to underlying principles of teaching and learning that constrain “what counts” as an example of a particular practice. At the same time, these principles also allow flexibility and innovation around talk routines, tasks, and tools that can foster student learning or participation.
• They are designed to be sensitive to the classroom context and to students' backgrounds and needs.
• They create and maintain equitable conditions for students from all backgrounds to engage in rigorous intellectual work related to the subject matter.

The grounding of teacher education in practice responds to two persistent challenges of professional preparation. The first, referred to as “the problem of enactment” (Kennedy, 1999), is the failure of teacher educators to help candidates implement ideas advocated by preparation programs in K-12 classrooms. Traditional preparation tends to focus on observing and analyzing classrooms and teaching, leaving translations from theory to practice in the hands of novices (Grossman & McDonald, 2008). The second challenge faced by teacher educators is the lack of consistency around the curriculum and the pedagogy of teacher preparation. There is no common curriculum for the preparation of teachers or best practices around the design of courses such as those focusing on instructional methods (Clift & Brady, 2005; Wilson, Floden, & Ferrini-Mundy, 2002). Data from a national sample of elementary science methods courses (Smith & Gess-Newsome, 2004) indicated that instructors chose course content based on self-selected objectives and perceived program needs. The authors concluded that, “what happens in methods classes depends largely on the beliefs and knowledge of the instructor” (p. 107). This is consistent with findings in other subject matter areas that describe how teacher candidate learning is not informed by research as much as the past experiences, skills, and worldviews of teacher education faculty and cooperating teachers (Ball et al., 2009; Deussen, Coskie, Robinson, & Autio, 2007; Little, 1990). There is little evidence that these preparatory experiences draw upon the respective knowledge bases of how K-12 students or pre-professionals learn.

A national report on preparing teachers could not find documentation to describe how methods courses are structured or the effects of these courses have on novice teachers' work in schools (National Research Council, 2010). Other studies have concluded that there are no best practices around the design of courses such as those focusing on instructional methods (Clift & Brady, 2005; Wilson et al., 2002). Wilson et al. (2002) found some evidence that preparation in pedagogy (e.g., courses in instructional methods, learning theories, and classroom management) could improve teachers' practice and students’ learning, however the research had not yet specified which elements of these experiences yielded positive outcomes. The practice-based movement in teacher education seeks more transparency about preparation and consistency in how teachers learn to do the work. Along the way, we hope to develop and refine shared language about instruction, to improve core practices themselves, and to help teacher educators advance their own pedagogy.

### 1.2 Sensemaking discussions as core to learning and promoting equity in science classrooms

For both issues of sensemaking and equity, the ability to orchestrate quality discourse has become one of the defining characteristics of effective teaching (Mercer, 2000; Michaels, O'Connor, & Resnick, 2008). There is broad consensus that structured opportunities to talk in classrooms support student learning in a variety of ways (Resnick, 2017). In classrooms, student talk has been used for sensemaking about science concepts (Hogan, 1999; Mortimer & Scott, 2003), negotiating the ‘rules of engagement’ around the use of claims and evidence (Herrenkohl, Palincsar, DeWater, & Kawasaki, 1999; Lehrer & Schauble, 2005), and positioning young learners as competent knowers of the natural world (Brown & Ryoo, 2008; Engle & Conant, 2002). The role of who is empowered to talk and the type of communication that is accepted as a part of science sensemaking is also an important consideration for creating equitable science learning experiences. How teachers engage students, such as asking classmates to compare their ideas, encouraging students to unpack their reasoning in a supportive environment, and allowing them to comment on their current state of understanding, can lead to deeper engagement in the content (Atwater, 2000; Duschl & Duncan, 2009) and to participation by young learners who would otherwise remain on the margins of classroom activity (Ballenger, 2009). When optimized, classroom discourse promotes students' funds of knowledge as a means for making sense of material activity in the classroom, helping better shape learners' identities as those who do science (González et al., 2005).

The idealized form of this instructional practice further addresses issues of equity within the classroom in multiple ways. Students' ideas are central to the meaning making and thus, even partial understandings are treated as resources for use in the building of collective understanding. Furthermore, students' conceptions are formed from a variety of sensemaking repertoires and sensemaking discussions have the potential to draw on multiple ways of expression that reflect cultural values and ways of knowing. And when conversations turn toward more canonical interactions, the teacher has the opportunity to explicitly define how and why scientists engage in particular exchanges, provide students the supports to participate safely, and make connections between individual talk preferences and those established by the scientific community (Windschitl et al., 2018). Importantly, within this paper we focus on teachers’ ability to create opportunities for equitable voice and access to building collective understanding that respects the cultural and historical lives of students in the classroom. While it is essential for teacher candidates to address the broader political and historical inequities that exist as a part of society and schooling (Zeichner, 2012), this paper focuses on only one aspect of one course within teacher training. Thus, it is beyond the scope of this paper to detail how the formation of these teacher candidates addresses the broadest and most systemic issues of equity.

### 1.3 Theoretical lenses on novice teacher learning

Teachers' instructional practices – such as selecting and setting up tasks, providing resources (e.g., tools, information, scaffolds) for students' engagement, or facilitating small group or whole group discourse – create conditions for groups of students to participate in disciplinary practices valued by the scientific and classroom community (Greeno & Gresalfi, 2008). In particular, creating robust discourse environments involves complex relational performances with young learners. To support these goals, novices develop their own visions of instructional excellence which are influenced by exposure to the instructional practices of others, heuristics acquired from early attempts at the work, and feedback from peers and teacher educators. In this process, teacher educators may provide conceptual tools (Grossman, Smagorinsky, & Valencia, 1999) or share their own curricular vision (Kennedy, 2006) that guides high-level decision-making about students’ interactions, and that contribute to particular learning goals. Teacher educators may also provide practical tools (Grossman et al., 1999), such as strategies or routines that can be directly used for planning and enactment. Ideas or practices from the preparation experiences become one of a body of resources that novices can draw upon to plan and enact instruction (Horn, Nolen, Ward, & Campbell, 2008; Nolen, Horn, Ward, & Childers, 2011).

Novices learn to do this work in a variety of ways. Learning can occur analytically through video study or explicit modeling by and ensuing discussion with the teacher educator. In contrast, the more passive apprenticeship of observation (Lortie, 1975) has been shown to shape novice teacher's beliefs and practices, sometimes with mixed results depending on the quality and alignment of the cooperating teacher's practice with the conceptual framework of the teacher education program (Stroupe, 2016). More actively, coaching and feedback can occur within methods courses as parts of rehearsals (Davis et al., 2017) or throughout supervised teaching in the classroom. In this paper, we focus on teacher educator modeling and rehearsal as pedagogies that can introduce, decompose, and provide opportunities for critique and feedback of complex, relational instructional practices. For clarity, we use ‘pedagogies’ to distinguish teacher educators' ways of interacting with teacher candidates from “practices” used by classroom teachers with students.

Cycles of learning framed by teacher educator modeling and subsequent teacher candidate rehearsal begin with teacher candidates playing the role of students while engaging in the disciplinary thinking and scientific practices authentic to a science classroom. During this modeling, action can be stopped to draw attention to moves by the ‘teacher’ or entertain questions by the teacher candidates about why certain instructional choices were made, for example, “Why did you re-voice her idea in that way?” This form of modeling is one of a suite of teacher education pedagogies that combines analysis and enactment of practice for teacher candidates. These pedagogies and their decomposition necessarily require attention to both skill *and* the professional judgment required in a specific learning context and instance that reflects the needs of the learners. The candidates then prepare for their own approximation of that practice, referred to as a rehearsal, with guidance from the teacher educator. They then enact a practice or part of a practice under supportive conditions, with peers playing the role of “students”. At the conclusion of each rehearsal, teacher candidates are given feedback immediately and may engage in further reflection. The teacher candidates can then enact these practices in a “live” classroom (Benedict-Chambers, 2016; Kazemi, Ghousseini, Cunard, & Turrou, 2016; Kazemi et al., 2016).

Within this framework of practice-based teacher education, this paper investigates what elements of the practice were most often taken up or left behind in teacher candidate rehearsals and classroom discussions. We consider uptake in terms of not only the technical skill, but the judgment and attention to context in carrying out particular instructional moves. Sawyer (2004) refers to this as “disciplined improvisation”. As excellence in teaching develops over time, we focus our analysis less on the novices' quality of enactments, and more on identifying connections between a teacher educator's pedagogies and what the teacher candidates attempt.

## 2 Method

### 2.1 Participants

This multi-site case study investigated the uptake of the core instructional practice, facilitating sensemaking discussion, of eight teacher candidates nested within two science methods courses. The first (Matt) and fourth (Mark) authors collected data from their science methods course for this study as part of a coordinated, multi-disciplinary data collection effort of the Core Practice Consortium (CPC) (corepracticeconsortium.com). The two teacher educators designed and implemented their practice-based methods courses independently of each other. Both methods courses included elements of teacher educator modeled instruction, reflection and analysis of the practice, support for teacher candidates’ own development of a teaching segment, and rehearsals. As part of the CPC, the two educators agreed to implement a general framework for leading sensemaking discussions that they helped co-design with other science teacher educators in the Consortium. Hence, certain commonalities specific to teaching the practice of facilitating discussion were expected. Below, we describe the two focal teacher educators and the programs in which they are situated. Then we provide brief descriptions of the eight teacher candidates.

**Matt's teacher educator context and methods course**. At the time of data collection, Matt was in his third year as an assistant professor at the University of Notre Dame. He had taught science methods courses for eight years, starting during graduate school. Prior to completing a doctoral degree in science education, Matt taught high school science and math for five years.

Matt's methods course was part of the University of Notre Dame's M.Ed. program. Candidates in the course included prospective middle and high school science teachers as part of a two-year, cohort-based, alternative certification program. Teacher candidates previously completed undergraduate work in the science discipline in which they would be teaching; these candidates then served for 2 years as the teacher of record in Catholic schools in the United States, most often schools enrolling underserved populations. Over the course of the program, candidates took a series of three science-methods and assessment courses and also participated in a practicum experience concurrently with the first methods course.

Matt's first semester science methods course was structured around five core practices, one of which was “facilitating sensemaking discussions”. Each of the practices, including facilitating sensemaking discussions, were modeled by the teacher educator, as well as represented via videos and transcripts of teacher and student talk. Teacher candidates prepared and rehearsed a sensemaking discussion based on a given data representation related to one aspect of global climate change. Rehearsals occurred with four other teacher candidates as “students” as well as the teacher educator. Rehearsals lasted approximately 20 min, including interactive “pauses” (Davis et al., 2017) and a 5 min debrief at the end of the rehearsal.

**Mark's teacher educator context and methods course**. At the time of data collection, Mark held the rank of full professor and had been teaching science methods at the University of Washington for nineteen years. Prior to completing his doctorate, Mark taught middle school science for thirteen years and taught in a university science department for two years.

Data from this study was gathered from the University of Washington's master's-level certification program. Teacher candidates entered the program as a cohort and had completed an undergraduate degree in an area of science or engineering. The program extended across a calendar year and included increasing levels of fieldwork in high-needs schools.

Mark's science methods course focused on a coordinated set of four core practices, one of which included the facilitation of sensemaking discussions. Candidates experienced the practice as learners as Mark modeled a discussion and made his thinking visible about key elements of the practice. Specific conceptual tools, such as a summary table, were also introduced as part of the practice and required for use by all rehearsing teacher candidates. Summary tables capture the collective understanding of students in relation to a preceding phenomenon or material activity. The summary table allows teachers to record agreed upon patterns, causal explanations, and connections to other phenomena. Similar to the rehearsals conducted at Notre Dame, candidates at the University of Washington practiced and received feedback on their facilitation of discussions with three other “students” and the teacher educator. However, whereas Matt's students rehearsed using given data sets, Mark's students rehearsed facilitating a discussion after “students” had engaged in material activity designed by the rehearsing teacher candidate.

**Teacher candidates.** Four teacher candidates, Bill, Erica, Mary, and Penny (all candidate names are pseudonyms) were part of Matt's eighteen-person Science Methods I course. All four candidates had undergraduate degrees in either biology or chemistry, and none had prior teaching experience. Similarly, four teacher candidates, Doug, Ricardo, Kelly, and Mandy were part of Mark's thirteen-person science methods course. All four candidates also had undergraduate degrees in the sciences and had no formal prior classroom teaching experience.

### 2.2 Data sources

**Teacher educator data**. Teacher educators captured multiple artifacts of the pedagogies for teaching the practice of facilitating sensemaking discussions. Data included: 1) syllabi and lesson plans, 2) class resources such as handouts, readings, and presentation slides, 3) pre-/post-methods course interviews exploring the goals, intentions, and reflections on teaching science methods and core practices in general and 4) methods class videos focused on teaching the practice of facilitating sensemaking discussions. It is important to note that the teacher educator data was purposefully bounded to the elements of the syllabus specifically targeted at teaching the practice of facilitating sensemaking discussions. In both university contexts, elements contributing to productive discussions, such as the introduction of “talk moves” or the elicitation of students’ ideas, were introduced at other points in the methods course. While these parts of the course influence the facilitation of sensemaking discussion, they were excluded from analysis.

**Teacher candidate data.** Each of the eight teacher candidates collected two videos of their practice: 1) a rehearsal of the candidate leading a sensemaking discussion that occurred during the methods course and 2) a classroom discussion with students in a “live” classroom context several months after the completion of the initial science methods course.

### 2.3 Data analysis

**Teacher educator framework.** We coded data from both teacher educators' artifacts and videos into two independent frameworks for teaching the practice of facilitating sensemaking discussions. The two frameworks – results in themselves – then served as the lens and codebook for analyzing the teacher candidates’ uptake in rehearsals and classroom discussions. We first coded the teacher educator data along four common components of facilitating sensemaking discussions that were specified by the entire science group of the Core Practice Consortium: 1) Framing the discussion, 2) Facilitating the discussion, 3) Publicly representing data and/or student ideas, and 4) Closing the discussion. These categories and their definitions were the explicit elements that the teacher educators helped co-design and sought teacher candidates to take up through their training. The categories of “Goals for sensemaking discussion” and “Equity considerations”, also served as initial parent categories for both sets of teacher educator data as these were discussed in the CPC and central to the foundational principles of the CPC and its core practice work.

Using this initial framework, two authors independently coded each piece of teacher educator data and entered instances of the parent code into an Excel spreadsheet. The coders also had the opportunity to add new parent level codes that extended beyond the initial framework. Upon completion of coding, the two coders addressed each piece of evidence, reconciled whether they agreed or disagreed about the entry for the parent code or the presence of a new parent code and created a “master” copy of the organized data.

Based on the nature of the data, codes were also marked as “Implicit (I)” or “Explicit (E)“. Implicit codes related to moves or elements of the practice that were modeled in the video, but never explicitly addressed in methods class discussion or materials. Explicit codes related to any data that appeared in a reading, the syllabus, or a handout that specifically addressed the construct. Furthermore, elements from the video in which the teacher educator explicitly told or discussed with teacher candidates the elements of practice, during a pause in modeling or during a debrief, were also marked as explicit. The authors who served as teacher educators did not code their own class's data. The research team then grouped parent and child codes and agreed upon common language (see Table 1 in Results).

**Table 1:** Coding frameworks for Matt and Mark's teaching of facilitating sensemaking discussion. Parent codes are lettered and child codes are numbered. The center column displays common parent and child codes while the far left and far right column display unique codes to each teacher educator. Implicit (I) and explicit (E) elements are also marked.

Matt	Common	Mark
3 Move from initial ideas toward understanding more canonical explanations of science. (E)	A. Goals for Sensemaking Discussion 1 Use and work with students' ideas about science. (E) 2 Build bridges between everyday language and formal science language. (E)	4 Make collective sense out of science activities. (E) 5 Develop productive relationships and identities with and among students. (E)
3 State interaction norms for working with each others' ideas. (I) 4 Make the discussion goal explicit. (E) 5 Clarify the stance toward the role of evidence in the discussion. (I)	B. Framing the Discussion 1 Make participation structures explicit. (I) 2 Convey norms of desired language use during the discussion. (E)	6 Identify norms for what types of ideas or comments are represented in the summary table. (E)
3 Provide no/minimal validation of student responses. (E)	C. Facilitating the Discussion 1 Use talk move prompts (elaboration, adding on to, disagreeing with, pressing for evidence, asking for clarification). (E) 2 Engage multiple students – not just the teacher – in an idea. (E)	4 Pre-select some ideas to start the discussion. (I)
	D. Representing Data or Ideas Publicly 1 Represent the teacher's own ideas publicly. (E) 2 Make decisions about who, when, and what is publicly represented. (E)	3 Use new knowledge to revise public representations of ideas. (E)
2 Prompt students to close the discussion (in whatever form). (E)	E. Closing the Discussion 1 Acknowledge remaining questions. (I)	3 Re-state shared understanding or ideas. (I)
	F. Equity Considerations 1 Construct welcoming discursive space. (E) 2 Make ideas and activities accessible to all students and their experiences so that they can engage. (E)	3 Hold high expectations for students of all cultures and languages. (E) 4 Engage students in overcoming boundaries to participation. (I)
G. Pre-requisites for Quality Discussion 1 Elicit students' initial ideas. (E) 2 Conduct investigations or activities that can shape and influence ideas. (E) 3 Prepare central questions. (E)		
		H. Centrality of Models to Discussion 1 The development of the model is central to carrying out effective discussions. (E)
		J. Centrality of Tools to Discussion 1 Tools are necessary to conduct effective discussions. (E) 2 The summary table is a central tool. (E)
		J. Coordinated Set of Practices 1 Discussion exists within a specific scope of practices: a) Introducing ideas to reason with b) Engaging with data or observations c) Using knowledge to revise models or explanations
		K. Pacing 1 Discussion requires iteration of activity and sensemaking discussion (I) 2 Sensemaking discussion requires large amounts of time. (E)

**Teacher candidate analysis.** Teacher candidate rehearsals and classroom discussions were analyzed with the appropriate teacher educator framework from Table 1. Rehearsals occurred within the context of the science methods course. For Matt's students, the classroom discussion occurred with the teacher candidate then serving as the teacher of record under the supervision of a mentor teacher and university supervisor. Thus, Matt's teacher candidates had the chance to establish the norms and procedures for science class interactions at the start of the year. For Mark's students, class discussions took place during student teaching embedded in a classroom with a cooperating teacher and university supervision. Teacher candidates had been in the classroom for about six weeks when the discussions were conducted.

Two members of the research team independently coded the videos using TORSH Talent™. Each instance of a code was recorded and then normalized to a percent of the total codes for that particular candidate's enactment. Frequencies were normalized as the rehearsals and classroom discussions varied greatly in length among the teacher candidates and within a teacher candidate's two enactments. Some codes from the teacher educators' frameworks were not codeable. These uncodeable aspects focused on a teacher candidate's intentions that could not be captured without a high level of inference. For example, “holding high expectations for students” was not captured explicitly even though many, if not all, of the candidates held high expectations for students. All codes were reconciled to 100% agreement.

### 2.4 Limitations

The case study methodology used in this study does not allow or intend to make any causal claims. Rather, these cases provide insight into opportunities and obstacles for teacher candidate uptake, providing some evidence for the influence methods courses can have on the development of core instructional practices. Furthermore, the decisions about what data could be used for analysis could suggest the false notion that teaching sensemaking discussions occurs in one bounded set of methods courses. Both Mark and Matt talked about laying the foundation for elements central to the discussion throughout the methods course and it is assumed that other courses address issues relevant to facilitating a discussion. Choices had to be made about bounding the data considered for analysis, thus likely providing more conservative findings of what elements were addressed in the methods class and carried into rehearsals and classroom discussions.

Finally, it is important to note that this paper looks at teacher candidates with the assumption that growth occurs over time. Our coding scheme defined uptake in terms of attempted enactment without coding for the *quality* of enactment. The two teacher educators had experience as both classroom teachers and teacher educators. Thus, comparing the quality of practice of teacher candidates to the examples they saw modeled in content methods courses would fail to recognize the developmental trajectory of teaching. Future studies could follow individual teachers longitudinally not only to see whether elements initially taken up from the methods course remained, but also whether enactments represented high quality instances of facilitating sensemaking discussions, especially attending to broader commitments to equity.

## 3 Results

### 3.1 Research question 1: two frameworks for facilitating sensemaking discussions

Despite a history of collaborative work, the frameworks for the core practice of facilitating sensemaking discussions of the two teacher educators varied, at times, in terms of what was implicitly or explicitly addressed in their methods classes, which sub-components were addressed for each of the common categories of the practice, and which additional elements were central within their own science methods course (Table 1). The common elements previously defined as part of the CPC work were present and addressed explicitly, but differences arose most prominently in the teachers’ different emphasis on using tools that support the practice and the coherence of how the practice fits within the broader set of practices taught in the respective courses.

Mark and Matt both explicitly established the *goals for discussions* – using and working with students' ideas as the raw material for the discussion. They both emphasized that discussions must help build bridges between students' everyday language about phenomena and more formal science language. They differed slightly on the intended outcomes as Matt explicitly talked about how the ultimate goal of the discussion was to move students from their own initial ideas to more robust understandings that used evidence to support canonical explanations of science and Mark explicitly focused on making collective sense of science activity that could then be used to revise models and existing representations. In doing this, he saw that students’ identities could be shaped, stating in a practice primer distributed to candidates that, teachers can, “use discourse for its full range of productive purposes, that is, for building and reinforcing productive identities and relationships”.

Mark and Matt commonly addressed *framing the discussion* – how “students” should participate in the whole group talk. For instance, the teacher educators established norms for acceptable language (i.e., whether to use scientific vocabulary or more common language) when they modeled a discussion and when they explicitly debriefed it. Matt also implicitly modeled specific ways in which “students” were expected to participate: 1) the norms for working with each other's ideas and how those ideas would be respected and 2) the privilege of evidence-based comments during the discussion. Most distinct from Mark, Matt stressed in both the classroom handouts and during the modeling and the modeling debrief that the teacher must frame the goals of the discussion for students. Meanwhile, Mark's framework uniquely focused on the relationship between the type of language and ideas that would be added to the summary table tool (Table 1).

Matt and Mark both modeled and discussed key components of *facilitating sensemaking discussions* that could be used to move students to engage in productive talk. These talk moves included pressing for evidence, re-voicing a student's idea, asking students if they wished to add on or disagree with other students' ideas, or asking students to elaborate on their own comment. Most importantly, and stressed by both teacher educators was the importance of engaging multiple students in the development of an idea. That is, teachers need to help students, often through the use of talk moves, to take up other students' ideas and work with them. This set of interactions contrasts an environment in which one student interacts with the teacher or multiple students serially to provide a stream of new ideas with each turn of talk. As part of this facilitation, Matt also explicitly talked about the importance of providing no or minimal initial validation of students' ideas in order for them to be taken up and discussed by other students. Mark uniquely modeled for candidates, but did not explicitly address, the importance of pre-selecting some students' ideas to seed the whole-class discussion.

In combination with the facilitation moves, Mark and Matt both explicitly stressed the role that *representing ideas publicly* can play in shaping a discussion. They discussed how students' attention is often drawn to what gets written on the board or other public places, and that discretion has to be used in deciding what to record. Both teacher educators modeled and discussed explicitly two forms of representations – representations of the teacher's own ideas and representations that result from the teacher making decisions about what student ideas should be publicly represented.

The two teacher educators differed on how they taught *closing the discussion* (Table 1). In Mark's classroom, the close was never addressed explicitly. Implicit messages were conveyed during the modeled discussion segment that included Mark's acknowledgement of remaining questions from the discussion and also Mark restating the shared understandings that had taken shape in the summary table. In contrast, Matt explicitly debriefed multiple ways in which the students would be responsible for closing the discussion in various forms – summarizing ideas, acknowledging remaining questions, or identifying areas of agreement or disagreement.

*Equity considerations* were embedded in the Core Practice Consortium's specification of this and other core practices. For the practice of facilitating classroom discussion, the teacher educators had outlined prior to the methods course the need to build on students' own language and experience in authentic, meaningful contexts. Throughout the experience, students' use of academic language requires the support of scaffolds and explicit structures for engaging in science talk. Furthermore, the type of discourse opportunities centered on open-ended questions in which students were treated as sensemakers, shifting the air time and power from the teacher to the student. These considerations were made evident in both teacher educator frameworks that included the intentional construction of a welcoming discursive space and making ideas and activities accessible to all students. Mark pushed this construct further as his written materials explicitly stated that during discussions, expectations must be held high for students of all cultures and language backgrounds. Implicitly, he also modeled how to help students overcome barriers to participation.

Evidence from Mark's science methods course also indicated that discussion was situated within a broader set of coordinated practices for teaching science (as described previously in Windschitl, Thompson, Braaten, 2008; Windschitl et al., 2012). Of note, the summary table tool was emphasized across the written handouts and was a central component of Mark's modeled discussion. While Matt's positioning of sensemaking discussions was less structured than Mark's, he explicitly identified several prerequisites for quality discussion, including the elicitation of students' initial ideas and the use of evidence from activity that could shape these ideas (Table 1). Finally, although the term “tool” was never used in his methods class, he cited the importance of developing central questions as the guide for meeting the goals of the sensemaking discussion. Thus, both teacher educators used and promoted tools for developing the practice, but Mark presented these tools in a much more structured and explicit way and focused more on tools to use throughout the enactment whereas Matt focused on tools that would help in the planning of the discussion, such as the identification of central questions.

### 3.2 Research question 2: “connecting the dots” from teacher education to candidate practice

**Fig. 1:**
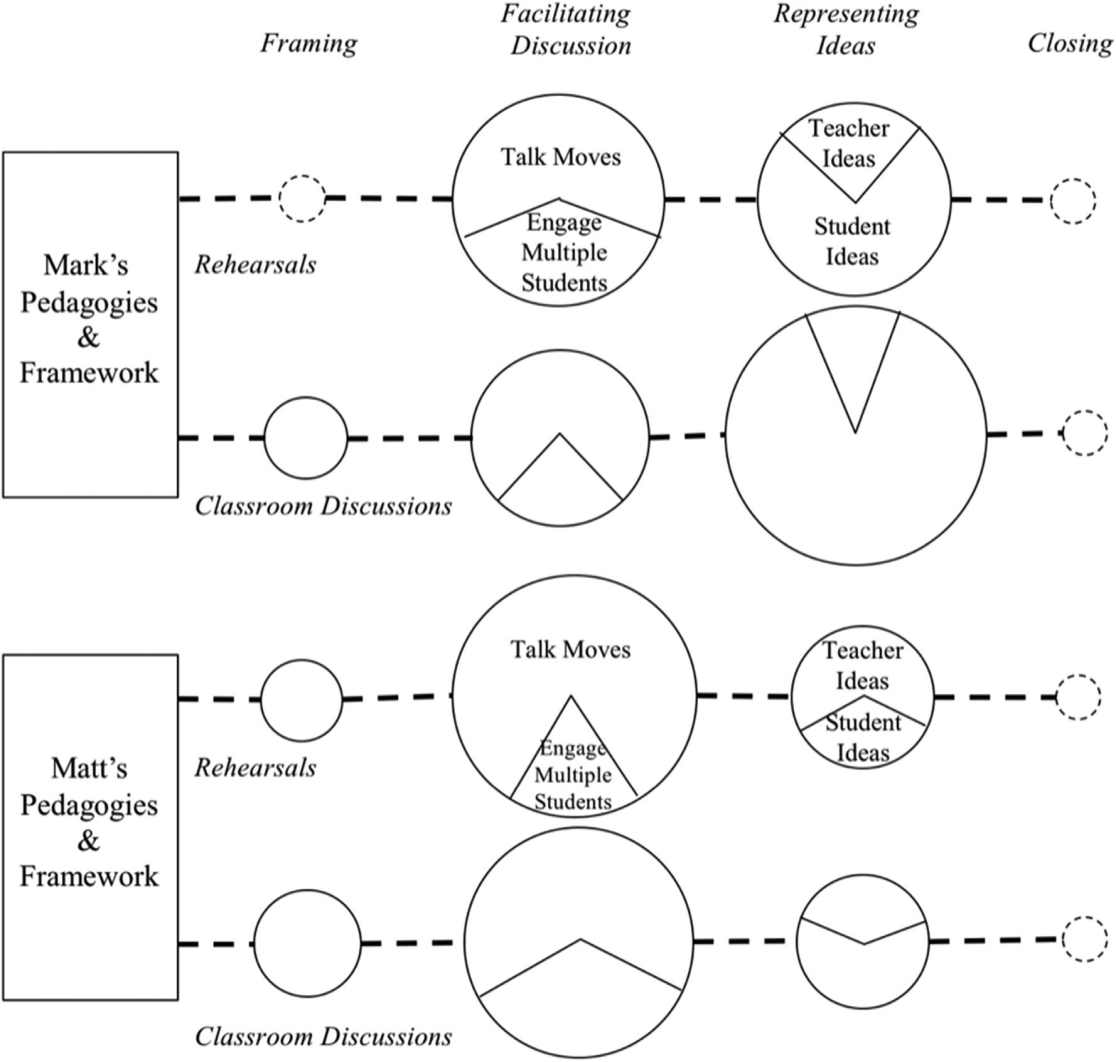
A summarized schematic of the main trends connecting teacher education pedagogies to teacher candidate uptake in rehearsals and classroom discussions. Dotted lines indicated minimal to no presence. Circle size represents proportion of moves for each of the four categories. Sections within circles represent trends and not exact proportions.

In this section we report findings that trace what occurred in the methods courses to teacher candidates' facilitation of sensemaking discussions first in rehearsals with peers and then in classroom discussions with students. Fig. 1 summarizes the major trends for Mark and Matt's candidates. Generally, three major trends appeared. First, the bookends of the discussion – the framing and the close – were sporadically taken up by candidates and often missing from rehearsals or classroom discussions. Second, candidates from both classes took up the talk move tools to engage students in the discussion, although the candidates' ability to engage multiple students in taking up a single idea was less consistent. Finally, the use of a specific tool – the summary table – led to consistent and high-levels of deciding what ideas to represent publicly for Mark's students, but Matt's students did not have this structure and invested less in representing ideas publicly.

**Fig. 2:**
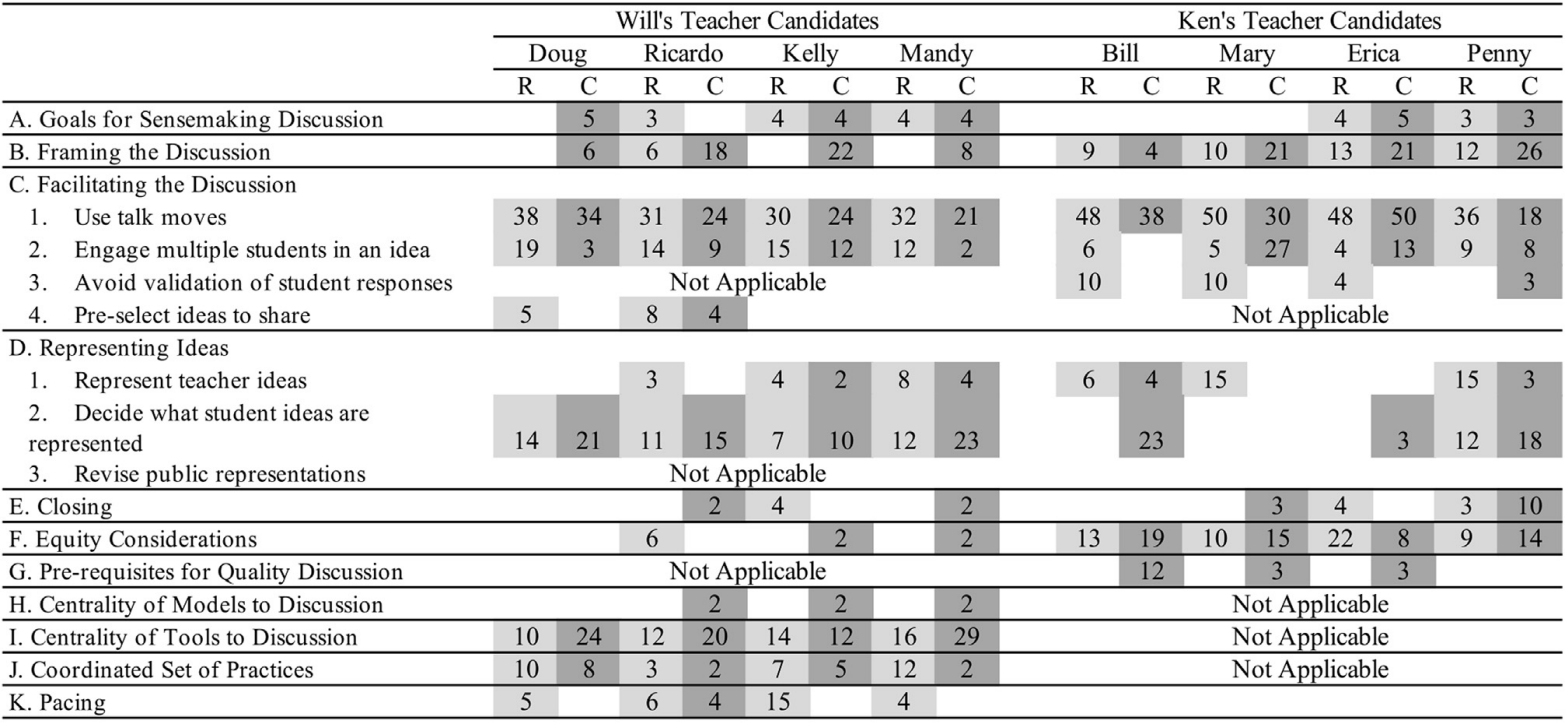
Connections between science methods courses, rehearsal approximations, and classroom enactments of facilitating a sensemaking discussion. Shaded boxes indicate whether a teacher candidate was coded for a particular component at least one time. Lighter shading represents rehearsals and darker shading represents classroom discussions. Numbers within cells represent the percentage that a particular component makes up of a teacher candidates set of codes within either their rehearsal (R) or classroom discussion (C). Child codes have been collapsed for all parent codes except for facilitating discussion and representing ideas.

Fig. 2 below represents a more granular perspective of what elements teacher candidates took up from the teacher educator frameworks, both in terms of rehearsals and classroom discussions. Each element from the framework is represented with light grey shading representing the rehearsals and dark grey shading representing the classroom discussions. Values inside each box represent the proportion of moves within a rehearsal or classroom discussion that each code represents.

**Mark's teacher candidate uptake.** The following data describe Mark's candidates' rehearsals and classroom discussions that both directly followed students engaging in material activity. Students were made aware that the *goal* of the discussion was to make sense out of the previous task as Katie exemplified at the beginning of her rehearsal when she said, “We have to think back to what we [did] and how that helps us explain our [big] idea”. While the explicit framing of *goals* was taken up by many teacher candidates, most elements of *framing* were not taken up by Mark's candidates in the rehearsals, (Fig. 2). Specifically, only one teacher candidate, Ricardo, addressed a framing code during the rehearsals – stating the norms for language use. This framing occurred multiple times as Ricardo and the “students” took up the student-suggested word, “frictiony,” when trying to describe the interaction between an object that was pulled across multiple surfaces. In this instance, Ricardo encouraged “students” to use common language, and in this case an invented word, rather than precise technical language to describe the phenomenon in question.

Despite its relative absence during rehearsals, techniques for framing the discussion appeared consistently throughout all four teacher candidates' classroom discussions. They all addressed participation structures, that is, how the teacher candidates wanted students to interact with each other and the class. In a majority of these cases, this type of framing took on the form of classroom management, such as asking students to raise their hands before speaking or allowing others to take turns talking. In addition, three of the four candidates addressed language norms with statements like, “I want you to use your own words.” Two teachers, Ricardo and Kelly, explicitly addressed what types of ideas would be appropriate for the summary table. When one student offered a causal explanation for a phenomenon, Ricardo thanked the student for his comment, but redirected the conversation by saying, “In this section [the observations column of the summary table], I don't want you to talk about why or give me the reasons yet, I want you to talk about what you saw in the video itself.”

Across both the rehearsals and the classroom discussions, Mark's teacher candidates *facilitated the discussion* using talk moves and making the summary table central in representing ideas publicly. In both enactments, Mark's teacher candidates used talk moves (Windschitl et al., 2012; TERC, 2011) proportionally more than nearly every other code. These talk moves included pressing students for elaboration, evidence, clarification or inviting other students to add on or disagree with claims made publicly. When Mark had modeled the use of talk moves in the teacher education course, he often asked students to “say more” or asked if “students” agreed or disagreed. In one exchange, he re-voiced an idea stated by one “student” and then engaged a new student in the idea saying, “I think I hear two slightly different ideas … I see Walt nodding his head. Do you see those as different ideas?” Candidates attempted similar types of exchanges in the rehearsals. For example, Doug took a claim from a “student” about the force reading on a scale that had preceded the discussion. Doug then turned to another group and asked, “Did you guys get the same thing? I'm curious how you went about the experiment?” The extensive use of talk moves continued from the rehearsals into the classroom discussions representing anywhere from 21 to 34% of each of the candidates' total instructional moves.

In rehearsals, candidates were able to engage multiple students in talking about an idea promoted by another student. While not as extensive as the proportion of talk moves used, anywhere from 12 to 19% of a candidates' moves in the rehearsals resulted in engaging multiple students. However, in the classroom discussions this proportion shifted to 2, 3, 9, and 12% of the total moves. For example, while 34% of Doug's moves in rehearsals were the use of talk moves, only 3% of his coded segments engaged multiple students in taking up the same idea. On multiple occasions, he followed a student's comment with the question, “Does anyone agree or disagree?“, but often, this question was met with no response and Doug either recorded the idea in the summary table or elicited a new idea.

Amongst Mark's teacher candidates, facilitating the discussion was linked tightly to *representing ideas publicly*. In the methods course, Mark presented a summary table – the four column chart described above that collects commonly held understandings about observations and causal reasons derived from an activity – as a formal tool used to facilitate sensemaking discussions. During the rehearsals, the summary table was the central means for recording ideas. Like Mark's modeling in the methods course, the teacher candidates used the summary table after several “students” had taken up and worked with a single idea. Only after some negotiation was an idea recorded. In rehearsals, the summary table was used from a low proportion of 5% of Doug's moves to a high of 12% of Mandy's moves.

Within the classroom discussions, representing ideas publicly using the summary table occurred with greater emphasis. For example, almost a quarter of Megan's classroom discussion codes focused on the decisions she had to make about what to represent in the summary table. As part of her whole class discussion, students presented their findings from multiple mini-labs. Based on these presentations, Mandy then asked the class if they agreed with the group's findings before re-voicing the findings and modeling for students what they should write on their own summary table. Similarly, Doug's proportional use of the summary table increased from only 5% of his codes in the rehearsal to 24% of his codes in the classroom discussion, Ricardo's proportion increased from 6% to 20% and Mandy's codes increased from 12% to 29%.

When Mark modeled the close of the discussion in his methods class, he acknowledged remaining questions from their discussion. Then he summarized the main ideas reported in the summary table and the collective, generalized knowledge that students now possessed from the discussion. The elements of *closing the discussion* were taken up far less by the candidates than the facilitation or representation moves. Only one of Mark's four teacher candidates acknowledged questions in the rehearsal and classroom discussion and only one of the teacher candidates restated the main ideas taken from the discussion. The lack of a focus on a close may have resulted from the time constraints that all four of the teacher candidates experienced during the rehearsals, but coding of the classroom video suggested that the teacher ran out of time to continue the discussion on only one occasion.

In summary, the strongest elements of uptake from Mark's science methods course to the rehearsals and the classroom discussions focused on the act of facilitating the discussion through the use of talk moves and the conscious use of the summary table to record students' ideas publicly. Each of these components was addressed by all four of the teacher candidates in both contexts, although the classroom discussions saw a slight decrease in the proportional use of talk moves and engaging multiple students in an idea. This decrease was juxtaposed by the increased use of the summary table and the necessary decisions that had to be made by the teacher candidate about what was represented on the board. In contrast, the framing and closing did not have the same level of uptake in the rehearsals, but elements of making explicit the participation structures and types of language desired from students reappeared in the classroom discussions.

**Matt's teacher candidate uptake**. In Matt's methods course, he explicitly stated one *goal* of discussions as moving students' initial ideas toward more canonical science ideas. In rehearsals, candidates were asked to take two data representations related to global climate change and move “students” from the data to understanding part of this core science idea. To save class time, Matt established the rehearsal norm that the “teacher” and “students” would all operate under the norm that evidence-based claims were privileged and participants in the discussion should use common language alongside scientific language. Despite these established *framing* norms, three of the four teacher candidates still addressed explicit structures for participation and brought up norms for language use. With the exception of Bill, attention to framing increased in the classroom discussions. Some of this increased attention was managerial – making sure that students understood how they should participate and interact with each other. Furthermore, these three candidates addressed a move that was absent in the rehearsals, but was addressed explicitly in the rehearsal debriefs – making the goal of the discussion explicit to students. For example, whereas Penny did not explicitly state the goal in the rehearsal, in the first 5 s of her classroom discussion she said, “We are going to try to answer the question, why do astronauts experience weightlessness?“.

Similar to Mark's candidates, Matt's candidates invested much of their time *facilitating the discussion* in rehearsals using talk moves. But unlike Mark's candidates who moved between orchestrating the talk and making decisions about what would be recorded in the summary table, only one of Matt's candidates represented students' ideas publicly in the rehearsals. Three of the teacher candidates spent nearly 50% of their codeable units using talk moves to facilitate the discussion (e.g., “Why do you think that?“). Despite the significant use of talk moves during rehearsals, the number of candidates' moves that successfully engaged multiple students in a presented idea was minimal (e.g., “Do you agree or disagree with Pamela?“). The four teacher candidates used eight to ten times more talk moves with students than moves that furthered uptake of an individual student's idea by other students. For example, in the rehearsal, Bill used a dozen talk moves in which he re-voiced a “student's” idea or asked a “student” to elaborate on her previous statement, such as asking, “Why?” in response to a student's explanation. However, only twice did he facilitate the discussion in a way that pushed “students” to build on the idea that was put forth by a student.

Matt's candidates continued to use productive talk moves in the classroom setting, but for three of the teacher candidates, the proportion of use decreased moderately from the rehearsal episodes. Interestingly, more of the moves facilitating the discussion engaged multiple students in discussing a single idea. For comparison, in the rehearsal, Mary had 50% of her codes focused on talk moves and only 5% indicating students' uptake of another student's idea, whereas the use of talk moves dropped to 30% in the classroom discussion but the uptake of student ideas increased to 27%.

Research suggests that providing *equitable* opportunities for and encouraging participation in discussions is critical for the success of all students (Calabrese Barton & Tan, 2009; Nasir, Snyder, Shah, & ross, 2012; Ryu, 2013; Windschitl et al., 2012). In addition to the talk moves, the methods course provided candidates some basic strategies and moves for constructing a welcoming discursive space. While modeling a discussion, Matt called on a “student” who stated that the previous student had already stated his response. Matt replied, “your words are important too,” and then the initially reticent student added on to the idea. These strategies appeared across all four teachers in the rehearsals and the classroom discussion. For example, Penny opened her rehearsal with an activity that asked students to share what they knew about sea level rise. In the classroom discussion, she specifically followed one question with the statement, “You don't have to be right or wrong, just [go] with your gut instinct.” She reiterated this idea as students began to write down their ideas, stating, “Don't worry if your [ideas] are right or wrong, just what are you thinking at this point?” In so doing, she not only invited students' ideas into the discursive space, but also expressed that their ideas were important and respected.

While candidates from both teacher education programs made extensive use of talk moves, Matt's candidates did not *represent students' ideas publicly* in as frequent or consistent manner as Mark's candidates. In the rehearsals, only Penny captured students' ideas on the board and while Bill, Erica, and Penny all captured some student ideas publicly in the classroom discussion, these representations were almost exclusively lists on the board of what individual students stated prior to those ideas being discussed. For instance, Penny asked students, “Why do astronauts experience weightlessness.” Students then started sharing out ideas and she wrote down all of the ideas that students suggested without taking any singular idea and having other students work with the idea before moving on or before confirming that the idea was part of the class's collective knowledge.

Similar to Mark's teacher candidates, elements of *closing* the discussion were minimally present and sporadic. In connecting the dots from the methods course where the close was explicitly discussed in terms of “students have to close and summarize the discussion, not [the teacher]“, only Penny held students responsible for the close in both the rehearsal and the classroom discussion. In attempting to have students close the discussion in her classroom, Penny stated, “So, can someone connect all those pieces? Can you say it one sentence?” Perhaps understandably, given the 15-min timeframe for the rehearsals, two of the teacher candidates did not provide any type of close during the rehearsals. But the lack of focus on the close extended into the classroom discussion with only Mary and Penny holding students responsible for the close.

In summary, Matt's candidates engaged in minimal, but increasing amounts of framing from the rehearsal to the classroom discussion. Generally, they focused their time in both enactments using talk moves and little time representing ideas publicly. Within these two categories, the candidates increased the amount of times they engaged multiple students in an idea from the rehearsal to classroom discussion and of the few ideas they represented publicly, more were student ideas and not their own. Similar to Mark's candidates, the close was only minimally addressed in both the rehearsal and the classroom discussion. In looking at Fig. 2, it is important to note that Bill's codes trend differently than the other three candidates in terms of explicit goal setting, engaging students in multiple ideas in the classroom setting or the amount of decisions made to represent ideas. The data suggest that he had the lowest amount of uptake in terms of making goals explicit during the framing. While he used talk moves in both the rehearsals and discussions, his classroom discussions consisted exclusively of volleys back and forth between him and individual students without engaging multiple students in taking up an idea put forth by a classmate. Finally, in both the rehearsal and the classroom discussion he ran out of time, resulting in no closing moves.

### 3.3 Research question 3: uptake of implicitly and explicitly taught practice elements

To better understand how teacher candidates’ uptake may have been afforded or constrained by pedagogical decisions of the teacher educators, part of the analysis for both groups of candidates focused on the extent of uptake based on whether components of the practice were explicitly or only implicitly addressed in the methods course. In general, the teacher educators were explicit about the main themes they addressed, but when implicit themes appeared, uptake was minimal.

Few categories from either teacher educator were labeled as “implicitly” presented to students (see “(I)” labels in Table 1). These implicit categories occurred as part of modeled practice by the teacher educator, but were not formally addressed orally or in writing within the data corpus. Across the set of implicit codes, no more than half of the candidates enacted any single code, with one significant exception. Both Mark and Matt modeled language and instructions about how they wanted the “students” to participate in different elements of the discussion. For instance, Mark indicated during the modeling segment whether students should raise their hand as part of turn taking during the discussion, but this part of his practice was never formally addressed. Similarly, during Matt's modeling of facilitating a sensemaking discussion, he provided instructions for moving students in and out of a pair-share prior to eliciting their ideas. During rehearsals, Mark's teacher candidates did not take up this implicit part of his pedagogy, allowing the other teacher candidates to participate in unstructured ways. Three of the four teacher candidates in Matt's class did address participation structures during the rehearsal, but in a limited way by using a pair share in each instance. However, in classroom discussions, seven of the eight teacher candidates made explicit the ways in which they wanted students to participate in the discussion (e.g., Penny stated, “Talk to your partner first, then talk to me.“).

Two of the three codes under the “closing the discussion” category were labeled as implicitly presented to teacher candidates. The code for the teacher acknowledging remaining questions was only observed once across the eight teachers in the rehearsals and twice – by two different teachers – across the eight teachers in the classroom discussions. Similarly, the code for having the teacher restate the main ideas of the discussion, a code only applicable to Mark's teacher candidates, was not seen in the rehearsals and was observed in only one classroom discussion. The one explicit code in this category, making students responsible for closing the discussion – applicable only to Matt's teacher candidates – appeared to have slightly more, yet still minimal, uptake with two teacher candidates using this close in the rehearsals and two teacher candidates using this close in the classroom discussion.

## 4 Discussion

Teacher education is a major influence on novice teachers’ development of effective instructional practice. As novice teachers are brought into the community of practice, they view and experience the profession from many different angles – historically, psychologically, sociologically, and practically. The science methods course connects these different perspectives as teacher candidates address the specific cognitive, social, cultural, and organizational elements of their future classrooms. Exploring what teacher candidates take up from these science methods courses provides both an empirical foundation for what components are more or less accessible to teacher candidates and formative assessment for adjusting the pedagogies and experiences within particular methods courses to increase uptake. Although multiple studies have focused either on the pedagogies of content methods courses in practice-based teacher education (e.g., Abell, Bryan, & Anderson, 1998; Windschitl, 2003; Marion, Hewson, Tabachnick, & Blomker, 1999) or on how teachers enact core instructional practices with students (Franke, Kazemi, & Battey, 2007), few empirical studies link what teacher candidates take up from these content methods courses and then put them into practice in a local approximation and/or authentic classroom discussion.

This study contributes to the literature by following two sets of teacher candidates from different teacher education programs into the field. Beyond analyzing the practice of the eight teacher candidates based on general principles often taught in science methods courses, this paper links the explicit and implicit components specifically addressed in the two teacher education programs of focus. Several themes emerged from the data that will be discussed below including: a) the affordances of approximations as a bridge to classroom practice; b) the influence of discursive and representational tools on practice; and c) the tension of practice-based instruction to move teacher candidates beyond taking up purely technical skills at the expense of practice more informed by professional judgment to promote student sensemaking.

Analyzing shifts in uptake patterns from rehearsals to authentic classroom discussions raises interesting questions about the affordances and limitations of approximations for teacher candidates. Approximations give teacher candidates opportunities to slow down the learning and try out different elements of the practice in a low-stakes scenario (Davis et al., 2017; Kazemi et al., 2016; Lampert et al., 2013). These rehearsals usually occur in concert with modeling or other pedagogies by the teacher educator where self-reflection on the quality of practice compared to a more experienced educator can occur. Indeed, the rehearsals provided opportunities for teacher candidates to try out some of the unnatural acts of teaching that Ball and Forzani (2009) describe. Generally, the rehearsals appear to function as a bridge from the methods course to classroom practice. With a few exceptions, elements of practice enacted by candidates in rehearsals occurred in classroom discussions as well, whereas elements that were absent in rehearsals were only sporadically enacted in the classroom discussions. For example, Mark's teacher candidates experienced the movement from material activity into the sensemaking of that activity during the methods course while Matt's teacher candidates experienced the movement from their initial ideas to more canonical understandings in their methods course. When taken up by teacher candidates in the rehearsal, these elements also generally appeared in the classroom discussion. Even more apparent was Mark's modeling of talk moves, the summary table, and the explicit statements of creating a welcoming discursive space for students; these elements appeared across all of his teacher candidates' rehearsals and within their classroom discussions.

Importantly, our data provide at least some empirical evidence that even if an element of practice is not taken up in rehearsals, the use of the rehearsal pauses and debrief may provide the bridge to classroom practice. As described in the results, although most of Matt's candidates did not frame the rehearsal discussion in terms of making the goal of the discussion explicit, this was addressed in the debrief and three of the four candidates did make the goal explicit in the classroom discussion. The reappearance of these elements of the practice in live classrooms discussions could be due to the use of pauses, “rewinds”, and debriefs (Davis et al., 2017) or the absence in rehearsals and reappearance in classroom discussions could result from teacher candidates feeling constrained on time in the rehearsals and making an explicit decision to focus on other parts of the practice given the short amount of time allotted. This might be especially true for the lack of closing opportunities for students across the rehearsals. Further empirical research, perhaps creating experimental conditions in which teacher candidates are assigned different lengths of time, number of students, or boundaries, can identify optimal conditions for approximating fully the different components of the practice.

The absence of uptake in rehearsals and the reappearance of uptake in classroom discussions might also result from the necessary artificiality that is embedded within the rehearsals. Working with peers who cooperate fully and who already have a common understanding of acceptable participation norms means that some elements of practice can be overlooked in the rehearsals. When novices enter their own classroom, however, these elements of the practice become more central to a teacher's enactment. As seen especially in Mark's teacher candidates' rehearsals, the peer teacher candidates who acted as students engaged naturally in discursive practices that may not occur with young people in a classroom setting – at least early in a novice teacher's development. In the classroom discussions, many teacher candidates were unable to engage multiple students in an idea even though they had done so successfully during the rehearsal (see Fig. 2). Thus, tensions may exist in the roles that are played in rehearsals. In order to focus the approximation centrally on a particular core instructional practice, “students” are often told not to act out behaviorally or present resistance to the instructional objectives of the teacher. These instructions are helpful in preventing approximations that focus mostly on classroom management and not working with students' ideas. However, a novice teachers' development can be even more difficult in a classroom setting when first met by factors presented by real students, such as the interaction of engaging in content while addressing management issues. In these instances, novice teachers' professional judgment and response to specific contexts is stretched.

Comparing teacher candidates' uptake across the two programs highlights the important role that tools can play in the support of novice teacher uptake. Windschitl et al. (2012) have empirically shown that once novices accept a practice as effective for facilitating student learning and frameworks for activities and discourse are in place, then tools can be developed to aid the enactment of these powerful interactions. They found that merely presenting a set of core instructional science practices did not further uptake of practice. Rather, “practice appeared to be influenced by the collectively developed pool of instructional resources that were created by participants themselves … [and] the most prominent example of a participant-developed resource [was] the face-to-face tools” (Windschitl et al., 2012, p. 893). The use of tools created and adapted by novices provides space for teachers to move beyond what could be the mechanical execution of a series of moves. These tools support the difficult work of leading discussions by focusing the cognitive work and, at times, making explicit space for attending to context. Both discursive and organizational tools played a significant role in teacher candidate uptake for both the rehearsal and the classroom discussions. The organizational tool of the summary table starkly differentiated how teacher candidates represented ideas publicly between Mark and Matt's candidates. Furthermore, the most frequent element of Mark and Matt's pedagogies that was taken up by teacher candidates was the use of the talk moves to facilitate the sensemaking discussion. These discursive tools provided teacher candidates with a basic set of moves that potentially maintain the focus on students' ideas and resist reversion to IRE questioning patterns. Asking students to elaborate or clarify their ideas, pressing them for evidence, or asking other students to add on to or disagree with public statements encourages students' uptake of these ideas. Across both the rehearsals and the classrooms discussions, the talk move discursive tools were central to the enactments. However, the presentation of discursive tools, like talk moves must be understood in their relation to student engagement. That is, teacher candidates in this study showed that they could use the talk moves in rehearsals and in the classroom, but the use of talk move phrases did not always invite or result in students taking up other students' ideas and moving collective understanding forward. Future studies should investigate the relationship between how tools, like talk moves, are presented and practiced in methods courses to best encourage multiple students engaging with an idea.

The use of the organizational tool (i.e., the summary table) in Mark's methods course highlights the magnitude of the influence these tools can have on practice, as teacher candidates in Matt's course were not exposed to this tool. In Mark's course, decisions about what to publicly represent were connected almost completely with the use of the summary table. All of the teacher candidates in Mark's course used the summary table and captured collective understandings on the board in the form of patterns and possible causal explanations. And as teacher candidates moved into the classroom setting, they became even more reliant on the tool, increasing the number of representations they captured publicly. To be clear, this is not a judgment on the quality of the representations captured, nor do we argue that more extensive use of the tool indicates more or less effective practice. Rather, it appears that in the more difficult and unnatural situation of negotiating ideas with students, the teacher candidates relied even more on the tool that they had seen in their methods course, and then approximated in the rehearsals. In contrast, Matt's teacher candidates were not introduced to a formal tool for representations and the act of making decisions about which student ideas to represent publicly was mostly absent from rehearsals and then only addressed by two teacher candidates in the classroom discussions.

Finally, methods courses are intended to explicitly develop the understanding and practices of teacher candidates, and in this case, for practice-based teaching. In this study, both teacher educators identified *a priori* ambitious goals for discussion and attended to aspects of equity within the specification. The variety of ways in which the teachers across the two programs enacted the discussions suggests that the commitment to a general framework which can be used flexibly across contexts is possible. That said, the case of Bill emphasizes the need for on-going instructional development after the methods course. Bill's classroom facilitation of discussion reflects the technical use of talk moves during the discussion. But the use of those moves – largely revoicings – did not result in students engaging with each others' ideas in an on-going manner. As instructional practices are composed of moves, routines, knowledge, and professional judgment, it is possible for novices, especially, to focus solely on isolated technical skills and not actually engage in the core practice of facilitating a discussion. Thus, while a high-quality specification and pedagogies are necessary, they may not be sufficient for novices, highlighting the importance of on-going support and quality feedback and coaching in the early years of teaching.

The role of addressing elements of practice implicitly or explicitly might also affect how teacher candidates move beyond seeing elements of a core practice as merely of technical importance or situated within larger goals of ambitious instruction. Implicit messages about professional judgment or attention to equity might be conveyed through modeling segments that do not get fully addressed or unpacked, leaving teacher candidates to focus more intently on the moves themselves than what the moves do to support students’ learning needs. Indeed, in analyzing the artifacts from the two methods courses, eight different elements of practice were implicitly addressed. As possibly expected, patterns in the data suggest that implicit modeling may be less influential on uptake with one exception – general issues of classroom management.

## 5 Conclusions

By “connecting the dots” between science methods courses, approximations, and classroom practices, we have shown how teacher candidates learn how to engage in the “unnatural act” of teaching (Ball & Forzani, 2009) by facilitating a sensemaking discussion. The case studies presented highlight how tools can support novice teachers' uptake of core instructional practices. Moreover, the findings serve as a reminder to teacher educators to explicitly address components of the practice to facilitate teacher candidates' understanding and uptake of these practices in both approximations and classrooms. Although we did not explore the quality of teachers' enactments of the elements of the practice, future research in teacher education could examine novices' developmental trajectories in their classrooms. Additionally, future studies should investigate the relationship between novice secondary science teachers' use of discussions and student learning. In so doing, we can continue to connect the dots and better understand which elements of the practice remain over time and how teachers’ use of core instructional practices can shape student learning.
